# Inter-laboratory validation of the measurement of follicle stimulating hormone (FSH) after various lengths of frozen storage

**DOI:** 10.1186/1477-7827-8-145

**Published:** 2010-11-29

**Authors:** Jessica Scriver, Valerie L Baker, Steven L Young, Barry Behr, Lisa M Pastore

**Affiliations:** 1 School of Medicine, University of Virginia, Charlottesville, VA 22908-0712, USA; 2 OB/GYN Department, Stanford University, CA 94305, USA; 3 OB/GYN Department, University of North Carolina, Chapel Hill, NC 27599-7570, USA; 4 OB/GYN Department, University of Virginia, Charlottesville, VA 22908-0712, USA

## Abstract

**Background:**

Serum follicle stimulating hormone (FSH) levels are used clinically to evaluate infertility, pituitary and gonadal disorders. With increased frequency of research collaborations across institutions, it is essential that inter-laboratory validation is addressed.

**Methods:**

An inter-laboratory validation of three commercial FSH immunoassays was performed with human serum samples of varying frozen storage length (2 batches of 15 samples each) at -25 degree C. Percentage differences and Bland-Altman limits of agreement were calculated.

**Results:**

The inter- and intra-laboratory consistency of FSH values with the same assay manufacturer was much higher after shorter-term storage (frozen for less than 11 months, mean percentage degradation less than 4%) than after long-term storage (2-3 years, mean percentage degradation = 23%). Comparing assay results from different manufacturers, there was similar overall long term degradation as seen with the same manufacturer (-25%), however the degradation was greater when the original FSH was greater than 20 mIU/mL relative to less than 10 mIU/mL (p < 0.001 trend test).

**Conclusion:**

The findings suggest that degradation of serum samples stored between 11 months and 2-3 years at -25 degrees C can lead to unstable FSH measurements. Inter-laboratory variability due to frozen storage time and manufacturer differences in assay results should be accounted for when designing and implementing research or clinical quality control activities involving serum FSH at multiple study sites.

## Background

Follicle-stimulating hormone (FSH) is a glycoprotein dimer secreted by the adenohypophysis that stimulates gametogenesis. Because serum FSH levels provide insight into the functional capacity of the hypothalamus, pituitary, and ovary or testis, they are frequently used for clinical evaluation of infertility, and disorders of the hypothalamic-pituitary-gonadal axis [1]. Specifically, FSH levels can be used to evaluate the etiology of low sperm counts, amenorrhea, menstrual irregularities, pituitary disorders, precocious or delayed puberty, and ovarian/testicular dysfunction [2,3].

Clinical determination of FSH concentration is typically measured in the serum, while measurement of urine FSH levels has replaced those in serum for some research protocols to avoid venipuncture. The correlation between urine and serum FSH values ranges from 70% - 90% [4].

Stability of stored FSH samples has been evaluated primarily in urine, with optimal storage conditions for sustained FSH immunoreactivity determined to be 1-4 weeks at 4°C due to significant degradation after 4 weeks [5]. Stability of immunoreactive urinary FSH seems most sensitive to time, with substantive declines (about 40%) after 50 weeks at -20°C [6] and 64 weeks at either 4°C and -20°C [7]. Storage of urine FSH samples at either room temperature or -20°C for only 1 week resulted in only a slight (2.8%) diminution of immunoreactivity [5]. Longer storage times (at least 1 year) can be successfully used if glycerol is added to the urine sample or the sample is extracted with acetone [7].

There is an absence of literature describing the temporal stability of immunoreactive FSH in frozen samples. Interestingly, a report examining the variability of serum FSH measurements between six different immunoassays concluded that the serum values were significantly different between different immunoassays. These authors speculated that the variability might be due to differential recognition of distinct FSH isoforms by each immunoassay [8]. However, that report was limited by the fact that their samples were stored at -70°C for an indeterminate amount of time, and the length of storage time was not addressed in their analysis.

While inter-laboratory validity of gonadotropins has not been well studied, inter-laboratory validity has been well-characterized for many other hormones, particularly sex steroids. For example, McShane, et al. demonstrated that the inter-laboratory coefficient of variation in the measurement of androstenedione, dehydroepiandrosterone sulfate, estrone, and estrone sulfate were all greater than 15%, resulting in large absolute differences in measured steroid concentrations [9].

Precise and accurate measurements of serum FSH levels are important for clinical and research purposes. The reduction of inter-laboratory variability becomes even more critical in collaborative, multi-center research projects [10]. Therefore, it is important to have meticulously standardized procedures for obtaining data measurements to ensure that the data collection is valid among different labs involved in a joint research endeavor.

Currently, there are no published studies using current assays that examine FSH stability in frozen storage. In addition, validation of inter-laboratory measurements of serum FSH has not been studied extensively, though studies tend to use FSH values obtained in various laboratories within an institution as interchangeable. In this study, we evaluated FSH measurements of the same sample in different labs after various lengths of storage time. The aims of this study were to determine how frozen storage time affects the validity of the FSH serum samples and to assess inter-laboratory variability.

## Methods

### Hormone measurements

Validation assays were performed in three separate laboratories: Stanford Reproductive Endocrinology and Infertility Lab (Laboratory A), University of Virginia Main Clinical Lab (Laboratory B), and University of Virginia General Clinical Research Center Core Lab (Laboratory C).

### Subjects

All serum samples were from female patients of reproductive age who presented to the Fertility and Reproductive Medicine Center at Stanford University. Patients signed an IRB-approved consent form, which allowed storage of their serum for research purposes. IRB approval was obtained and patient confidentiality was protected in this study. Chronologically, this study commenced with 15 human female serum FSH samples that were originally drawn during 2006-2008, all during the early follicular menstrual phase (i.e., generally cycle days 2-5). This series of samples is subsequently termed the "Long Term Batch".

For the second part of the study ("Short Term Batch"), serum FSH samples were obtained from a different group of 15 women, all in the follicular phase and all in 2009.

### Specimen handling

For the Long Term Batch, 15 stored samples were chosen in order to represent a range of FSH values and initially run in Lab A. Blood samples were obtained by venipuncture, and serum was separated from the cells by centrifugation in Lab A. On the same day as the blood draw, the samples were analyzed and then frozen in Lab A. The samples were de-identified and assigned a number. These samples were stored at -25°C for a mean of 2.3 years. Six samples were stored for three years, eight samples were stored for two years, and one sample was stored for 1 year. None of the lab freezers used in this study were "frost-free" and the storage temperature was stable.

The samples from the Long Term Batch were sent to Labs B and C with sequential coding (sample numbers 1-15) without any information on the original assay results. The samples were taken out of the freezer at Lab A after their respective lengths of frozen time and shipped overnight to Lab B on dry ice. Upon arrival at Lab B, the samples were immediately thawed and the immunoassays were run the same day. These samples were then driven over to Lab C on the same day, and the samples were analyzed at Lab C without undergoing another freeze-thaw cycle. Each sample was only run a single time at each lab.

For the Short Term Batch, the second 15 stored samples were again initially obtained as described above, and run in Lab A. These samples were analyzed and frozen at -25°C on the same day that the samples were obtained. The samples remained frozen for 1-2 months (mean = 45.27 days, range = 30-69 days) and then they were thawed and re-assayed at Lab A. These Short Term Batch samples were sent to Labs B and C with sequential coding (sample numbers 16-30) without any information on the original assay results. The samples were shipped overnight with cold packs to Lab B on the same day as the second Lab A assay in order to avoid an additional freeze/thaw cycle. The next morning, upon arrival at Lab B, the immunoassays were run. Again, these samples were driven to Lab C on the same day, and the samples were analyzed at Lab C later that day without undergoing another freeze-thaw cycle. Therefore, these samples were all originally processed in Lab A, and then re-run in all three Labs after 1-2 months of frozen storage at -25°C. All of these samples were only run a single time in each lab and all measurements were recorded.

The samples from the Short Term Batch were subsequently stored at Lab C for the next nine months at -20°C in order to obtain FSH measurements after an intermediate frozen storage time. After nine months, the samples were thawed under refrigeration until completely liquid, and then assayed immediately in Lab C. They were then refrigerated until transport, driven to Lab B in a cooler with frozen ice packs, and then assayed in Lab B on the same day.

### Assay characteristics

At Lab A, the samples were run on an Immulite 2500 Automated Immunoassay Analyzer. This is a solid-phase, two site chemiluminescent immunometric assay. 200 beads, coated with monoclonal murine anti-FSH are used. The analysis is based on an alkaline phosphatase label and a chemiluminescent substrate that uses a centrifugal wash method. Three controls were run with each assay and 2 out of 3 of the controls must be within 2 standard deviations for the run to be accepted. The coefficient of variation (CV) for each control level was as follows: I (mean ≅ 5) = 3.73, II (mean ≅ 15) = 4.6, and III (mean ≅ 35) = 3.76.

At Lab B, the samples were run on a fully automated Abbott Architect ci8200. The Architect FSH assay uses a two-step chemiluminescent immunoassay technology that incorporates an acridinium derivative tracer. The CV for each control level was: I = 2.49, II = 2.65, and III = 2.54.

At Lab C, the samples were run on an Immulite 2000 Automated Immunoassay Analyzer, which is a fully automated, continuous random access instrument. The Immulite 2000 has 24 reagent positions, and uses the same reagent and bead formulation as the Immulite 2500. As true for the Immulite 2500 from Lab A, 3 controls were run with each assay and 2 out of 3 of the controls must be within 2 standard deviations for the run to be accepted. The CV's in Lab C were: I = 5.6, II = 6.3, and III = 3.6.

### Statistics

For each batch of samples, lab, and storage timeframe (short term, intermediate, and long term), the summary mean, standard deviation, median, standard errors and quartile were tabulated. The results were analyzed with percentage differences to explore the impact of assay manufacturer and/or time length of frozen storage. Both mean and median percentage differences were calculated. Only the mean percentage differences are displayed due to the close similarity between the results.

Assay measurement agreement was analyzed in accordance with the Bland Altman method [11,12] by way of random effects analysis of variance (ANOVA) and by way of two-way ANOVA. For the two-way ANOVA, the Welch version of the Student's test was utilized, because the group variances were not equal and this method does not require equal variances in order to obtain valid tests of statistical inference. Statistical significance was measured with an alpha = 0.05.

## Results

### Short term storage - intra-lab

Serum FSH results on the day of the original blood draw (Lab A only) and after short-term storage of approximately 45 days (all labs) are shown in Table 1 and are all based on the Short Term Batch samples. The values range from normal to modestly elevated for a woman of reproductive age. Comparison within Lab A between the immediate results and those after 45 days indicate the impact of short term frozen storage. The mean degradation was a minimal 4% in this scenario (Table 2, Comparison 1).

**Table 1:** Human female serum FSH (IU/L): assay summary statistics after less than 12 months frozen storage (short term and intermediate storage times)

**Lab**_**storage time**_	Mean (SD)	Median (SE)	Min, Max	Q25, Q75
Lab A_0 days_	7.31 (3.29)	7.60 (0.85)	2.20, 14.50	5.15, 9.05
Lab A_45 days_	7.00 (3.02)	7.40 (0.78)	2.10, 12.90	4.70, 8.90
Lab B_45 days_	6.07 (2.57)	6.47 (0.66)	1.95, 10.53	4.20, 6.72
Lab C _45 days_	7.29 (3.01)	7.92 (0.78)	2.18, 12.80	5.00, 9.12
Lab B_10.5 months_	6.33 (2.79)	6.60 (0.72)	2.10, 11.40	4.55, 7.05
Lab C_10.5 months_	7.29 (2.96)	7.79 (0.76)	2.31, 12.00	4.95, 9.22

n = 15 samples

SD standard deviation, SE standard error, MIN minimum value, MAX maximum value, Q25 25^th ^percentile, Q75 75^th ^percentile.

**Table 2:** Human female serum FSH (IU/L): assay to assay comparisons after varying length of storage time at -25 degrees C

Comparison	Labs and storage time	Mean Percentage Difference (SD)
1	A_45 days _vs. A_0_	-4% (0.05)
2	A_45 days _vs. C_45 days_	+5% (0.03)
3	A_0 days _vs. C_45 days_	+1% (0.07)
4	A_45 days _vs. B_45 days_	-12% (0.08)
5	A_0 days _vs. B_45 days_	-16% (0.09)
6	B_45 days _vs. B_10.5 months_	+4% (0.05)
7	C_45 days _vs. C_10.5 months_	0% (0.04)
8	A_0 days _vs. C_2 yrs_	-23% (0.13)
9	A_0 days _vs. B_2 yrs_	-25% (0.20)

SD standard deviation

### Short term storage - different lab with same assay manufacturer

**Figure 1:**
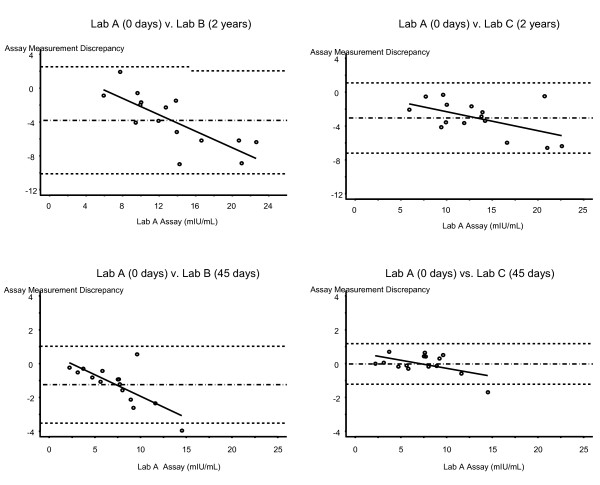
**Assay measurement agreement by batch and paired labs**. For each graph, the difference between the FSH (mIUm/L) values between two labs is plotted on the y-axis, and the x-axis represents the mean Time 0 Lab A assay value. The degree of agreement is estimated by calculating the bias, which is represented by the horizontal dashed lines at the mean ± 2 standard deviations. Each graph contains a comparison between two labs in one batch of samples.

Both Lab A and Lab C use an instrument by the same manufacturer for the FSH assay. Thus, comparison of Lab A and Lab C results after the identical short term frozen storage time indicates variability between this manufacturer's FSH instruments. In this instance, the variability is small (5%, Table 2, Comparison 2). Comparison of the Lab C results after 45 days of storage to the Lab A results run on the day of the venipuncture (Table 2, Comparison 3) shows a 1% difference, thus reinforcing that that there is little degradation of human serum FSH in the short term when the lab instrument manufacturer variable is held constant. The Bland-Altman limits of agreement are -1.2 mIU/mL to +1.2 mIU/mL for Lab A and Lab C under this short-term storage scenario. Figure 1 (bottom right plot) illustrates the tight standard deviation band; the fact that the band is centered on zero reinforces the minimal degree of degradation in this comparison. The degree of variability in these samples was larger at higher levels of FSH (p = 0.045, test for trend).

### Short term storage - different lab and different assay manufacturer

The FSH instruments used by Labs A and B are from different manufacturers. Comparison of the Short Term Batch results from Lab A and Lab B after the identical short term frozen storage time indicates variability by manufacturer in addition to potential differential impact by storage time. In this instance, the variability is moderate (-12%, Table 2, Comparison 4). Comparison of the Lab B results after 45 days of storage to the Lab A results on the day of sample collection (Table 2, Comparison 5) shows -16% degradation, thus implying that the variability may be due more to differing manufacturers than the frozen storage time, as this difference is similar to Comparison 4. The Bland-Altman limits of agreement are -3.5 mIU/mL to +1.0 mIU/mL for Lab A and Lab B under this short-term storage scenario, which is a wider band of agreement than between Lab A and Lab C. Figure 1 (bottom left plot) illustrates the wider measurement discrepancy band relative to the "same manufacturer" comparison above. The degradation in these samples was greater at higher levels of FSH (p = 0.002, test for trend). The mean percentage degradation for samples originally less than 10 mIU/mL was 15% vs. 24% for samples ≥ 10 mIU/mL.

### Intermediate storage time - intra-lab

Serum FSH results were also evaluated among the Short Term Batch after intermediate-term storage time by comparing the 45-day values for Short Term Batch in Labs B and C vs. the re-run values in Labs B and C after an additional frozen storage period of nine months (Table 1 and 2). Within both labs, the mean FSH level after the additional time in frozen storage was minimal: +4% in Lab B (Table 2, Comparison 6) and 0% in Lab C (Table 2, Comparison 7).

### Long term storage - different lab and same assay manufacturer

The impact of long term frozen storage time was evaluated with the Long Term Batch (Table 3). Comparing the serum FSH level in Lab A on the day of venipuncture with the re-run value from Lab C after 1-3 years of -25°C storage indicates a significant degradation occurred in this timeframe (-23%, Table 2, Comparison 8). As these labs use the same assay manufacturer, this degradation is likely due to the frozen storage time rather than variability between the labs. The Bland-Altman limits of agreement are -7.2 IU/L to 1.1 IU/L for Lab A and Lab C under this long-term storage scenario. The degree of degradation in these long-term-storage samples varied by level of FSH (p = 0.036, test for trend, Figure 1 top right). Although that test was statistically significant, the difference in the mean long term percentage degradation by FSH tier was clinically minimal: 25%, 23% and 21% for samples originally ≤ 10 mIU/mL, 10 mIU/mL to 20 mIU/mL, and > 20 mIU/mL, respectively.

**Table 3:** Human female serum FSH (IU/L): assay summary statistics after long term (median 2 years) storage at -25 degrees C

**Lab**_**storage time**_	Mean (SD)	Median (SE)	Min, Max	Q25, Q75
Lab A_0_	13.33 (5.02)	12.70 (1.30)	5.90, 22.60	9.75, 15.40
Lab B_1-3 years_	9.53 (3.27)	9.00 (0.84)	5.00, 16.20	7.95, 11.25
Lab C_1-3 years_	10.27 (4.26)	10.60 (1.10)	3.80, 20.20	7.69, 11.25

n = 15 samples

SD standard deviation, SE standard error, MIN minimum value, MAX maximum value, Q25 25^th ^percentile, Q75 75^th ^percentile.

### Long term storage - different lab and different assay manufacturer

FSH levels in Lab B after long-term storage (Long Term Batch) are shown in Table 3. The mean percentage difference between the results from Lab A on the day of the blood draw and the re-run values from Lab B after 1-3 years of frozen storage was -25% (Table 2, Comparison 9). The similarity of the degradation in Comparisons 8 and 9 implies that frozen storage time, even at -25°C, has a greater impact on serum FSH degradation than variability between assay manufacturers. The Bland-Altman limits of agreement are -10.1 to 2.5 for Lab A and Lab B under this long-term storage scenario, meaning that the actual absolute difference between measurements in Lab A and Lab B is between 10.1 IU/L and -2.5 IU/L. As shown in Figure 1 (top left), the degree of degradation in these samples was larger at higher levels of FSH (p < 0.001, test for trend). The mean long term percentage degradation was 12% for samples originally ≤ 10 mIU/mL vs. 31% for samples between 10 mIU/mL and 20 mIU/mL vs. 34% for samples > 20 mIU/mL. Note the wider standard deviation bands in Figure 1 for both Long Term Batch graphics (top 2 plots) relative to the Short Term Batch graphics (bottom two plots).

### ANOVA calculations - storage time and immunoassay

Two-way ANOVA concluded that the difference in the storage length (as measured by comparing the two batches) was statistically significant (p < 0.001), while the difference in the labs was not statistically significant (p = 0.066).

## Discussion

In summary, these findings indicate that (a) almost all of the serum FSH measurements after long-term storage at -25°C are less than the original values with an average degradation of 25%, and (b) a shorter storage time (less than 11 months) at -25°C showed much less decline in immunoreactivity than samples stored for an average of two years. Also, almost all the serum FSH measurements that used the same assay manufacturer (Labs A and C) were similar when the frozen storage time was less than 11 months. Taken together, these results imply that frozen storage time has a large impact on the degradation of serum FSH samples, while the specific immunoassay does not appear to be as important of a factor in explaining the variation in the FSH values. This conclusion is supported by the ANOVA results.

The inter-laboratory variation increased directly with increased frozen storage time at -25°C. This trend was observed irrespective of which laboratory or immunoassay was used. The similarity in the FSH sample measurements after frozen storage times of 4-9 weeks and 10.5 months suggests that serum FSH samples are stable through ten months. While the Short Term Batch and the Long Term Batch represent different serum samples, these data strongly suggest that serum FSH samples do degrade to unacceptable levels after 2-3 years of frozen storage at -25°C. The degree of degradation (-25% between Labs A and B, -28% between Labs A and C) after long term frozen storage are unacceptable for research purposes, with the possible exception of studies of postmenopausal women where any value > 40 mIU/mL might be sufficient. The degree of agreement after short-term storage in the Short Term Batch is markedly improved (less than 5% variation using the Labs A and C assay manufacturer).

The results indicate an interesting difference between assay manufacturers in term of the degradation by FSH level. These data imply that the long term degradation is greater at higher levels of FSH with the Abbott Labs machine (Lab B) more so than with the Immulite machine (Labs A and C). This is an area for future research, as our short term batch had no samples over 15 mIU/mL and neither batch had any samples greater than 25 mIU/mL.

A strength of this study is the use of three different laboratories for the assays and two distinct batches of samples, stored for different lengths of time. The use of multiple laboratories served to model collaborative research. Furthermore, comparing two different labs that are part of the same institution was helpful for replicating discrepancies that can occur even within an institution. Having two different batches of samples frozen for different amounts of time allowed investigation of the impact of storage time while limiting the freeze-thaw cycles that could confound the interpretation of the measurements, unless the original samples were initially aliquoted into multiple subsamples.

Limitations of this study include a small sample size and limited population. There were only 30 total samples in this study, limiting the precision of our conclusions. However, this study can serve as a pilot for establishing variables for future research on this topic. The population of samples was limited to women attending a fertility clinic, some of whom will have higher than average FSH samples for reproductive age women. It is possible that the sample degradation may be different for samples with average FSH values versus samples with elevated FSH values; the trend test results imply that possibility. The range of FSH measurements in the Short Term Batch was narrower than in the Long Term Batch: the range of Lab A FSH values was 2.2 - 14.5 mIU/mL in the Short Term Batch versus 5.9 - 22.6 mIU/mL in the Long Term Batch. Thus, the samples do not equally reflect women with elevated, pre-menopausal levels of FSH.

It may also be beneficial in a future study to take the average of multiple measurements for each sample. In this study, we only measured each sample once for each measurement, and this may have allowed for some inaccuracies because each of these assays has an inherent variability. In addition, this study had a complex design with multiple levels of variation in both the length of frozen storage time and assay manufacturer. Further studies could be conducted to look at these variables independently.

This study serves to raise important considerations when using samples from different sites and the effect of frozen storage length. Areas for future research include freezing samples for various lengths of time in order to determine the degree of degradation as time progresses in samples stored for longer than 10 months. Additionally, other temperatures of storage could be investigated. All these samples were stored at -25°C, but other storage temperatures may possibly maintain the sample integrity longer. Urine FSH samples are ideally stored at 4°C [3], and there is a high correlation between serum and urine FSH samples, so the temperature of storage for serum FSH samples needs to be explored more in order to determine ideal storage conditions.

In addition, variability may also be related to individual laboratory techniques or human lab technique variability. Each laboratory's procedure in this study used a different immunoassay. Therefore, as has been suggested in previous research on FSH reliability, it may be useful to have standardized immunoassays within a research protocol [8].

FSH measurements have important clinical implications for medical management as well as for entry criteria into studies. Due to the increasing trend towards having multiple clinical sites collaborate on research studies in order to increase sample sizes, it is now essential to ensure that there is acceptable reliability of the FSH measurement across research labs. This study served to demonstrate that frozen storage time primarily and assay manufacturer secondarily may lead to decreased reliability of the measurements of human serum FSH samples.

## Competing interests

The authors declare that they have no competing interests.

## Authors' contributions

JS wrote the article. VLB provided study samples, provided input on the interpretation of the results, and edited the manuscript. BB conducted some of the lab assays and edited the manuscript. SLY provided input on the interpretation of the results and the data analysis, and edited the manuscript. LMP designed the study, conducted some of the statistical analysis, and supervised the writing and statistical analyses. All authors read and approved the final manuscript.
